# A Computational Study of Executive Dysfunction in Amyotrophic Lateral Sclerosis

**DOI:** 10.3390/jcm9082605

**Published:** 2020-08-11

**Authors:** Alexander Steinke, Florian Lange, Caroline Seer, Susanne Petri, Bruno Kopp

**Affiliations:** 1 Department of Neurology, Hannover Medical School, Carl-Neuberg-Straße 1, 30625 Hannover, Germany; florian.lange@kuleuven.be (F.L.); caroline.seer@kuleuven.be (C.S.); petri.susanne@mh-hannover.de (S.P.); kopp.bruno@mh-hannover.de (B.K.); 2 Behavioral Engineering Research Group, KU Leuven, Naamsestraat 69, 3000 Leuven, Belgium; 3 Movement Control & Neuroplasticity Research Group, Department of Movement Sciences, KU Leuven, Leuven, Tervuursevest 101, 3001 Leuven, Belgium; 4 LBI-KU Leuven Brain Institute, KU Leuven, 3000 Leuven, Belgium

**Keywords:** executive dysfunction, computational modeling, reinforcement learning, amyotrophic lateral sclerosis, Parkinson’s disease, Wisconsin Card Sorting Test

## Abstract

Executive dysfunction is a well-documented, yet nonspecific corollary of various neurological diseases and psychiatric disorders. Here, we applied computational modeling of latent cognition for executive control in amyotrophic lateral sclerosis (ALS) patients. We utilized a parallel reinforcement learning model of trial-by-trial Wisconsin Card Sorting Test (WCST) behavior. Eighteen ALS patients and 21 matched healthy control participants were assessed on a computerized variant of the WCST (cWCST). ALS patients showed latent cognitive symptoms, which can be characterized as bradyphrenia and haphazard responding. A comparison with results from a recent computational Parkinson’s disease (PD) study (Steinke et al., 2020, J Clin Med) suggests that bradyphrenia represents a disease-nonspecific latent cognitive symptom of ALS and PD patients alike. Haphazard responding seems to be a disease-specific latent cognitive symptom of ALS, whereas impaired stimulus-response learning seems to be a disease-specific latent cognitive symptom of PD. These data were obtained from the careful modeling of trial-by-trial behavior on the cWCST, and they suggest that computational cognitive neuropsychology provides nosologically specific indicators of latent facets of executive dysfunction in ALS (and PD) patients, which remain undiscoverable for traditional behavioral cognitive neuropsychology. We discuss implications for neuropsychological assessment, and we discuss opportunities for confirmatory computational brain imaging studies.

## 1. Introduction

The ability to maintain goal-directed behavior effectively is an important prerequisite for successful daily life, in particular in face of interfering information [1,2,3]. This overarching cognitive ability is often referred to as executive control [4,5]. The Wisconsin Card Sorting Test (WCST) [6,7,8] is considered as a gold standard for the neuropsychological assessment of cognitive flexibility, which represents an important facet of the broader concept of executive control [4,9,10,11].

**Figure 1:**
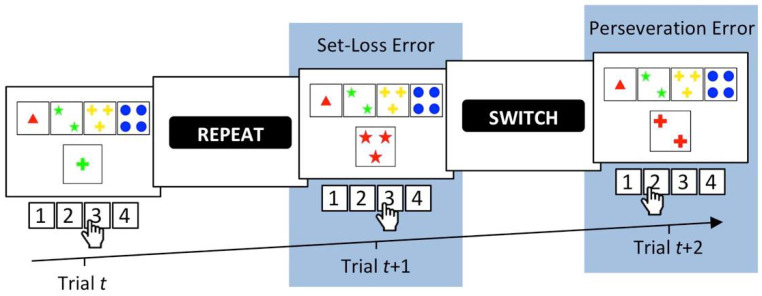
A showcase trial sequence on the computerized WCST [12,13,14] which was used in this study [15]. On Trial *t*, the stimulus card depicts one green cross. It could be sorted by the color category (inner left key card, response 2), the number category (far left key card, response 1), or the shape category (inner right key card, response 3). The observation of response 3 indicates that the shape category was applied. A subsequent positive feedback (i.e., “REPEAT”) indicated that this response was correct and that the shape category should be repeated on the upcoming trials. However, on Trial *t* + 1, response 3 was pressed, indicating that the number category was applied. Erroneous switches of the applied category following positive feedback are called *set-loss errors*. Next, a negative feedback (i.e., “SWITCH”) indicates that this response was incorrect, implying that a category switch is required. On Trial *t* + 2, the number category was erroneously repeated as response 2 was pressed. Erroneous repetitions of categories after negative feedback are called *perseveration errors*.

The WCST requires participants to sort stimulus cards to key cards by categories that change periodically, as detailed in Figure 1. In order to identify the currently prevailing category, participants have to rely on the examiner’s positive and negative feedback on each trial. Negative feedback on a WCST-trial requests switching the previously applied category, whereas positive feedback indicates that the previously applied category should be repeated. Of major interest for the present study are perseveration errors (PE), which refer to non-desirable category repetitions following negative feedback, and set-loss errors (SLE), which refer to unsolicited category switches following positive feedback [12].

Increased WCST error propensities (usually measured as enhanced PE and/or SLE rates; [16]) are well-documented neuropsychological corollaries of many neurodegenerative diseases [15,17,18,19,20]. Among these diseases—and of particular interest for the current study—is amyotrophic lateral sclerosis (ALS), which is characterized by a loss of upper and lower motor neurons in the brain and spinal cord neurons [21]. Accumulating research suggests that ALS pathophysiology comprises non-motor, prefrontal cortical areas in a non-negligible subset of patients [22,23,24,25]. As such, about 15% of ALS patients are affected by the behavioral variant of frontotemporal dementia [26,27,28]. Of further interest for the present study is Parkinson’s disease (PD), which is primarily characterized by a loss of dopaminergic neurons in nigro-striatal pathways [29,30]. Increased WCST error propensities were also observed in many other neurological diseases [31,32,33] and psychiatric disorders [34,35,36,37,38]. Therefore, the named behavioral measures of increased WCST error propensities do not provide sufficient nosological specificity to serve as pathognomonic neuropsychological symptoms of particular neurological diseases or psychiatric disorders [12,39].

The insufficient nosological specificity of WCST error propensities may relate to the ‘impureness’ of behavioral WCST measures [11,12,40,41,42,43]. That is to say that behavioral WCST measures may originate from a mixture of multiple covert cognitive processes. The dysfunction of any single cognitive process, or of any combination of these processes, could manifest itself as increased WCST error propensities [12]. These considerations suggest that WCST measures may be severely limited in their diagnostic utility as a consequence of their impurity at the level of covert cognitive processes.

Yet, increased WCST error propensities may still arise from potentially dissociable covert cognitive impairments in diverse patient groups. Such circumscribed cognitive impairments may not be detectable by WCST error analyses because they may *all* be behaviorally expressed as enhanced PE and/or SLE rates, as discussed above. As one example, both ALS patients and PD patients show increased error propensities on the WCST (see above). Despite this commonality between the two groups of patients, the neurodegenerative alterations that occur in ALS patients could still affect a set of covert cognitive processes that remain spared in PD patients, who, in contrast, show alterations in a different set of covert cognitive processes [12]. Thus, dissociable covert cognitive impairments might be detected across these (and other) groups of patients with pathophysiological characteristics that are at least partially distinct. This possibility remains viable despite the fact that it has been clear for a long time that the nosological specificity of the commonly considered behavioral WCST measures remains unsatisfactory [31,44,45].

Computational cognitive neuropsychology offers an approach to decompose behavior that was observed on neuropsychological assessment instruments into covert cognitive processes [41,46]. Computational cognitive neuropsychology utilizes mathematical formalization of (1) the assumed covert cognitive processes, and (2) the way in which these processes interact [47,48,49,50,51,52,53,54,55,56,57]. Analyzing behavior on a neuropsychological assessment instrument, such as the WCST, via computational modeling allows estimating a set of latent variables, which reflect the efficacy of the assumed covert cognitive processes. Computational cognitive neuropsychology yields covert cognitive variables across diverse patient groups, which may be more specifically related to some of the pathophysiological characteristics of neurodegenerative diseases than behavioral measures, such as WCST error propensities. The present study exemplifies how computational cognitive neuropsychology may contribute to advancements in the field of executive control by (1) providing novel WCST-based latent variables reflecting different facets of cognitive flexibility, and (2) analyzing their nosological specificity in patients with ALS in comparison with patients with PD.

Several previously published studies conducted computational modeling of behavioral performance on the WCST [42,58,59,60,61,62,63,64,65,66,67,68,69,70], but surprisingly none of these earlier studies applied reinforcement learning (RL) models [71,72,73,74,75,76,77]. RL represents a suitable framework for modeling WCST behavior because of the potential reinforcement-quality of WCST feedback stimuli [40,78] that were illustrated in Figure 1. The present study relies on a particular modification of RL, which we referred to as parallel RL in two related previous studies from our group [33,34].

Our parallel RL model [33,34] conceptualizes WCST behavior as resulting from two parallel, yet independent RL processes [40,79]. Cognitive learning at a conceptually higher level is complemented by sensorimotor learning at a conceptually lower level ([40] provides a rationale for the introduction of the two parallel RL levels). With regard to the WCST, cognitive learning considers objects of thought (i.e., which category to apply; cf. Figure 1) that guide the selection of task-appropriate responses: The reception of positive feedback enhances activation values of the currently applied category, whereas the reception of negative feedback reduces activation values of the currently applied category. Simple sensorimotor learning bypasses these objects of thought as it is solely concerned with selecting responses, irrespective of the to-be-applied category: Responses that were followed by positive feedback tend to be repeated, whereas responses that were followed by negative feedback tend to be avoided on upcoming trials.

**Figure 2:**
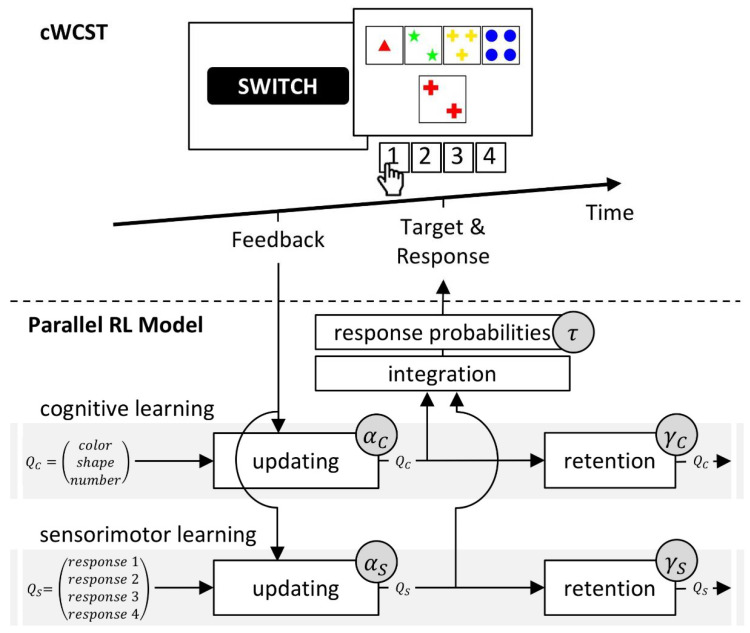
A schematic representation of the cWCST and the parallel RL model. A negative feedback on the cWCST (top) indicates that the previously executed response (not depicted) was incorrect, implying that a category switch is required. The subsequent stimulus card is sorted by the color category, as indicated by observing response 1. The parallel RL model (bottom) assumes independent trial-by-trial cognitive and sensorimotor learning (upper and lower grey bar, respectively). Core to cognitive and sensorimotor learning are feedback predictions for the application of categories ($Q_{C}$) and the execution of responses ($Q_{S}$), respectively. Feedback predictions are updated in response to received feedback. Individual cognitive ($\alpha_{C}$) and sensorimotor learning rates ($\alpha_{S}$), which are further separated for positive and negative feedback, quantify the strengths of updating. For the subsequent target, cognitive and sensorimotor feedback predictions are integrated. Response probabilities are rendered from these integrated feedback predictions, with an individual inverse temperature parameter (τ) quantifies how well response probabilities accord to integrated feedback predictions. Retention mechanisms ensure that feedback predictions transfer to the next trial. Here, individual cognitive ($\gamma_{C}$) and sensorimotor retention rates ($\gamma_{S}$) quantify the strengths of retention of feedback predictions.

The parallel RL model comprises independently variable learning rates for positive and negative feedback because learning from positive and negative outcomes seems to be supported by distinct brain systems [80,81,82]. Our parallel RL model hence quantifies individual learning rates for positive and negative feedback for both cognitive and sensorimotor learning as latent variables. Our parallel RL model also comprises two additional latent variables that are not inherent parts of canonical RL models [71]. First, separate retention rates at cognitive and sensorimotor levels [83,84] quantify how well previously learned information remained available on current trials. Second, a ‘soft-max’ function [71,85] expresses how well finally executed responses accorded to the information that had been learned from feedback; we refer to this latent variable as the inverse temperature parameter (see also [86,87]). Figure 2 gives a schematic representation of the parallel RL model.

In a recently published study [41], we characterized PD patients’ alterations in latent variables of the parallel RL model by studying PD patients ‘on’ and ‘off’ their clinically prescribed dopamine (DA) medication and a matched healthy control (HC) group on a computerized WCST (cWCST) [88]. Application of the parallel RL model revealed that PD patients showed enhanced cognitive retention rates and reduced sensorimotor retention rates compared to HC participants. We concluded that (1) enhanced cognitive retention rates express bradyphrenia [89,90,91,92], and that (2) reduced sensorimotor retention rates express disturbed stimulus-response learning [93,94,95] at the level of latent cognitive symptoms in PD patients. Systemic DA replacement therapy did not remedy these two anomalies in PD patients but incurred non-desirable side effects such as decreasing cognitive learning rates in response to positive feedback. Iatrogenic side effects of DA replacement therapy in PD patients may be due to an overdosing of meso-limbic and/or cortical DA systems [96,97,98,99].

The present study examined the specificity of executive dysfunction in ALS through (1) a computational analysis of WCST behavior in patients with ALS and (2) a comparison between their latent cognitive profile and that of patients with PD from an earlier computational study [41]. In order to achieve these goals, we characterized ALS patients’ alterations in latent variables of the parallel RL model [33] by studying ALS patients and a matched HC group on a cWCST variant [15]. Executive dysfunctions are among the well-known neuropsychological sequelae of ALS pathophysiology [15,17,100], which comprises—at least in a non-negligible subset of ALS patients—prefrontal cortical areas [22,23,24,25]. This led us to predict that ALS patients may show evidence for alterations in latent variables of cognitive learning, which is putatively supported by prefrontal cortical areas of the brain [41,79,101]. Overall, the present computational cWCST study was designed to gain information about the nosological specificity of alterations in latent variables of the parallel RL model in ALS patients compared to PD patients.

## 2. Materials and Methods

### 2.1. Participants

Twenty-one ALS patients were recruited from the ALS/MND clinic at Hannover Medical School. All ALS patients fulfilled the revised El Escorial criteria for clinically probable or definite ALS [102]. ALS patients who had a history of any neurological disease other than ALS or any psychiatric disorder were not considered for participation in this study. Furthermore, ALS patients who had highly restricted pulmonary function or were not able to press a button on the response pad due to motor impairment were not considered for inclusion in this study. We excluded two ALS patients because of invalid cWCST behavior. cWCST behavior was considered invalid if participants committed odd errors (i.e., responses that match no viable sorting category) on more than 20% of all completed trials. Note that odd errors on more than 20% of all completed trials could indicate that card sorting behavior did not accord to instructed categories. We also excluded one ALS patient who showed clinical signs of frontotemporal dementia [103], resulting in a final sample of 18 ALS patients. Seventeen ALS patients had limb-onset disease and one patient had bulbar-onset disease. None of the patients had a percutaneous endoscopic gastrostomy.

The healthy control (HC) group comprised 21 participants without any diagnosed neurological or psychiatric disorder. HC participants were age-, gender- and education-matched to the initial sample of ALS patients [15]. Table A1 in Appendix A shows ALS patients’ and HC participants’ demographic, clinical, and neuropsychological characteristics. Participants were offered payment as compensation for participation (30 €). The study protocol was approved by the local ethics committee (vote number: 6269) and was in accordance with the Declaration of Helsinki. All participants gave written informed consent. Note that an initial analysis of this data was reported by [15].

### 2.2. Computerized Wisconsin Card Sorting Test

Participants completed a variant of the cWCST [15]. Participants were required to sort stimulus cards to key cards *W* = {one red triangle, two green stars, three yellow crosses, and four blue balls} by one of three sorting categories *U* = {color, shape, number}. Card sorts were indicated by button presses *V* = {response 1, response 2, response 3, response 4} on a standard computer keyboard. Buttons were spatially mapped to key cards. Stimulus cards varied on the three dimensions color, shape, and number and never shared more than one feature with any of the key cards, rendering the applied sorting category unambiguously identifiable [104]. The target display presented the four key cards, which appeared invariantly above the stimulus card. 

Card sorts were followed by positive and negative feedback cues that were the German words for repeat (i.e., ‘bleiben’) or shift (i.e., ‘wechseln’), respectively. A positive feedback cue indicated that the executed response was correct and that the applied sorting category should be repeated on the upcoming trial. A negative feedback cue indicated that the executed response was incorrect and that the applied sorting category should be shifted on the upcoming trial [105]. On any trial, the application of only one of the three sorting categories was correct. 

The target display was presented until a button was pressed. Feedback cues remained on screen for 500 ms. The interval between a button press (i.e., target display offset) and feedback cue onset was 1500 ms and the interval between feedback cue offset and target display onset was 1000 ms. Participants completed a practice session of 15 trials prior to the experimental session. The experimental session consisted of a pseudo-randomly generated sequence of 120 trials. The correct sorting category switched after a variable number of trials (mean number of trials until a switch of the correct category was 3.8). Participants were explicitly informed about the three viable sorting categories and about the fact that correct sorting categories would change periodically. The experiment was programmed using Presentation^®^ (Neurobehavioral Systems, Berkeley, CA USA).

### 2.3. Error Analysis

We analyzed PE (i.e., erroneous repetitions of the applied category after negative feedback) and SLE (i.e., erroneous switches of the applied category after positive feedback) [12,40,41]. Conditional error probabilities were computed as the ratio of the number of committed errors and the number of trials on which a respective error type was possible (i.e., all trials following negative or positive feedback for PE and SLE, respectively). We analyzed conditional error probabilities by means of a Bayesian repeated measures analysis of variance (ANOVA) using JASP version 0.11.1 (JASP Team, Amsterdam, The Netherlands) [106]. The Bayesian ANOVA included the within-subjects factor error type (PE vs. SLE) and the between subjects factor group (HC vs. ALS).

We reported results of the Bayesian ANOVA as analysis of effects [107]. That is, evidence for an effect (i.e., a main effect of error type or group or the interaction of error type and group) in the data was quantified by inclusion Bayes factors (BF_inclusion_). Inclusion Bayes factors give the change from prior probability odds to posterior probability odds for the inclusion of an effect. Prior probabilities for the inclusion of an effect *P*(inclusion) were computed as the sum of all prior probabilities of ANOVA models that included the effect of interest. Posterior probabilities for the inclusion of an effect *P*(inclusion|data) were computed as the sum of all posterior probabilities of these ANOVA models. We used default settings of JASP for the Bayesian ANOVA as well as uniform prior probabilities for all ANOVA models. Descriptive statistics were reported as mean conditional error probabilities with 95% credibility intervals. We computed 95% credibility intervals as the interval of 1.96 standard errors of the mean around the mean.

### 2.4. Computational Modeling

The parallel RL model [40,41] is based on a conceptualization of cWCST behavior as parallel cognitive and sensorimotor learning. Cognitive and sensorimotor learning are operationalized by Q-learning algorithms [71,108,109,110,111]. The implemented Q-learning algorithms operate on feedback prediction values, which quantify the strength of prediction of positive (feedback prediction values larger than 0) or negative feedback (feedback prediction values less than 0) following the application of a specific category (for cognitive learning) or the execution of a response (for sensorimotor learning). On any trial, feedback prediction values are updated in response to an observed feedback (Equations (A3) and (A7)). How strong feedback prediction values are updated is modulated by prediction errors, which are the difference between the observed feedback and the current feedback prediction value (Equations (A2) and (A6)). Higher absolute prediction errors indicate stronger updating of feedback prediction values. Appendix B gives a mathematical description of the parallel RL model as well as details of parameter estimation.

The parallel RL model comprises cognitive and sensorimotor learning rate parameters. Learning rates quantify the extent to which prediction errors are integrated into feedback prediction values of the applied category (for cognitive learning; Equation (A3)) or the executed response (for sensorimotor learning; Equation (A7)). On a behavioral level, a high cognitive learning rate indicates that a participant strongly adapts category selection to received feedback (i.e., a participant tends strongly to repeat or switch an applied category following positive or negative feedback, respectively), whereas a low cognitive learning rate indicates a marginal adaptation of category selection to received feedback. Likewise, a high sensorimotor learning rate indicates that a participant strongly adapts the sensorimotor selection of responses to received feedback (i.e., a participant tends strongly to repeat or avoid the execution of a particular response following positive or negative feedback, respectively). In contrast, a low sensorimotor learning rate indicates that a participant barely adapts the sensorimotor selection of responses to received feedback. As we implemented separate cognitive and sensorimotor learning rate parameters for trials that follow positive and negative feedback, each participant is characterized by a set of four learning rate parameters, i.e., cognitive learning rates for positive ($\alpha_{C}^{+}$) and negative feedback ($\alpha_{C}^{-}$) as well as sensorimotor learning rates for positive ($\alpha_{S}^{+}$) and negative feedback ($\alpha_{S}^{-}$).

The parallel RL model also parameterizes retention rates [83,84] at cognitive and sensorimotor levels (i.e., $\gamma_{C}$ and $\gamma_{S}$, respectively). Retention rates quantify the extent to which feedback prediction values from the previous trial will transfer to the current trial [40,41,83,84] (Equations (A1) and (A5)). Higher retention rates indicate more pronounced transfers to current trials. At the cognitive level, higher retention rates should support adequate WCST behavior in response to positive feedback (attenuating SLE), but higher retention rates should interfere with adequate WCST behavior in response to negative feedback (enhancing PE). At the sensorimotor level, higher retention rates should lead to repetitive patterns of responding following positive feedback, as the resulting positive feedback prediction values retain higher levels of activation. In contrast, higher retention rates should lead to non-repetitive responding following negative feedback, as the resulting negative feedback prediction values retain larger (negative) levels of activation. Overall, retention rates reflect participants’ flexibility vs. stability [112,113,114] at cognitive or sensorimotor levels. With regard to successful cWCST behavior, high degrees of flexibility at the cognitive level are advantageous in response to negative feedback, whereas high degrees of stability are advantageous in response to positive feedback. 

Eventual card sorts on any trial arise from the integration of cognitive and sensorimotor feedback prediction values (Equation (A8)). The extent to which finally overt responses accord to these integrated feedback prediction values is quantified by an individual inverse temperature parameter τ [71,85] (Equation (A9)). A low inverse temperature parameter indicates that responses strictly accord to integrated cognitive and sensorimotor learning, whereas a high inverse temperature parameter indicates that card sorts are less dependent of integrated cognitive and sensorimotor learning. Thus, with a high inverse temperature parameter, responses appear to be more random.

We used Bayesian tests for direction to quantify evidence for group-related shifts of model parameters [108,115]. Bayes factors larger than 1 indicate evidence for an increase of a model parameter in ALS patients when compared to HC participants. Bayes factors less than 1 indicate evidence for a decrease in a model parameter in ALS patients when compared to HC participants. We interpreted Bayes factors by classes of evidential strength following [116]; Bayes factors larger than 10 (or less than 1/10) were interpreted as strong evidence for the presence of an effect, and Bayes factors larger than 100 (or less than 1/100) were interpreted as extreme evidence for the presence of an effect. This classification of Bayes factors was also used for the interpretation of results of the behavioral analysis. The implemented code of computational modeling analysis can be downloaded from https://osf.io/46nj5/.

## 3. Results

### 3.1. Error Analysis

**Figure 3:**
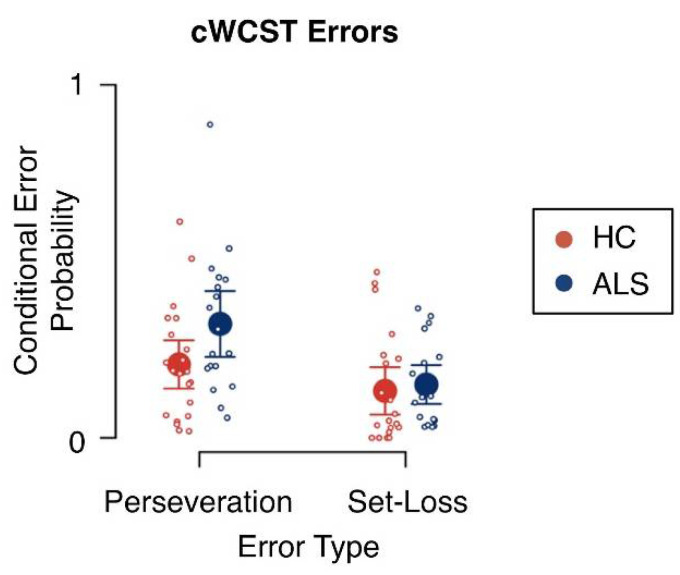
Conditional error probabilities. Large circles indicate mean conditional error probabilities. Error bars indicate the 95% credibility interval. Small circles indicate individual conditional error probabilities. HC = healthy control participants; ALS = amyotrophic lateral sclerosis patients.

Mean conditional error probabilities are shown in Figure 3 and results of the Bayesian ANOVA are reported in Table 1. There was extreme evidence for an effect of error type on conditional error probabilities (BF_inclusion_ = 836.42). Conditional PE probabilities (*m* = 0.26; *SE* = 0.03) were generally higher than conditional SLE probabilities (*m* = 0.14; *SE* = 0.02). There was no evidence for a main effect of group on conditional error probabilities (BF_inclusion_ = 1.27) and there was no evidence for an interaction effect of error type and group on conditional error probabilities (BF_inclusion_ = 2.50).

### 3.2. Computational Modeling

Cognitive learning rates were overall higher after positive (median = 0.48, IQR = 0.11) than after negative feedback (median = 0.06, IQR = 0.04), indicating that participants showed stronger cognitive learning after positive than after negative feedback. In contrast, sensorimotor learning rates were overall higher after negative feedback (median = 0.05, IQR = 0.02) than after positive feedback (median < 0.01, IQR = 0.01). In fact, the sensorimotor learning rate after positive feedback was virtually zero, indicating that participants showed sensorimotor learning after negative feedback, whereas no sensorimotor learning happened after positive feedback. Taken together, cognitive learning rates were overall higher than sensorimotor learning rates, indicating a generally stronger contribution of cognitive learning on cWCST behavior when compared to sensorimotor learning. The sensorimotor retention rate (median = 0.21, IQR = 0.23) was overall higher than the cognitive retention rate (median = 0.11, IQR = 0.12). Thus, participants retained more sensorimotor information from previous trials than cognitive information.

**Figure 4:**
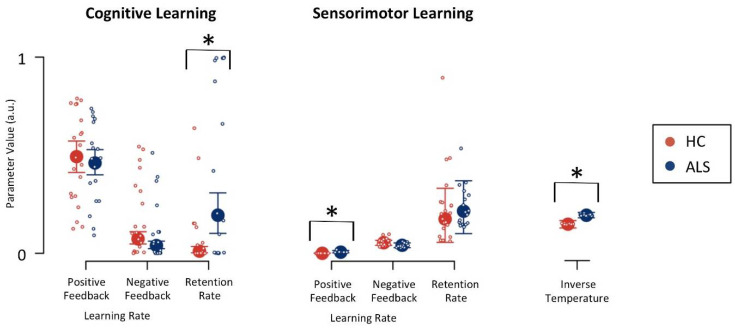
Model parameters for cognitive and sensorimotor learning. Large circles indicate medians of group-level posterior distributions. Error bars indicate lower and upper quartiles of group-level posterior distributions. Small circles indicate medians of individual-level posterior distributions; a.u. = arbitrary units; * strong evidence for the presence of a group difference; HC = healthy control participants; ALS = amyotrophic lateral sclerosis patients.

Figure 4 shows parameter estimates for ALS patients and healthy control participants. There was strong evidence (BF = 37.46; Table 2) for an increased cognitive retention rate in ALS patients (median = 0.19, IQR = 0.21) when compared to healthy control participants (median = 0.01, IQR = 0.03), indicating that ALS patients retained more cognitive-learning information from previous trials. There was strong evidence (BF = 17.07) for an increased inverse temperature parameter in ALS patients (median = 0.19, IQR = 0.03) when compared to healthy control participants (median = 0.15, IQR = 0.04), indicating an overall more random responding of ALS patients. Lastly, there was strong evidence (BF = 14.96) for an increased sensorimotor learning rate following positive feedback in ALS patients (median = 0.01, IQR = 0.01) when compared to healthy control participants (median < 0.01, IQR < 0.01).

## 4. Discussion

The present data demonstrate how specific facets of executive dysfunction in ALS patients may be investigated through the application of computational techniques. Computational modeling of cWCST behavior by means of the parallel RL model [40] revealed that ALS patients show increased cognitive retention rates when compared to HC participants, indicating that ALS patients retained more cognitive information learned on previous trials. Furthermore, ALS patients showed increased inverse temperature parameters when compared to HC participants, indicating that ALS patients’ actual responding was more independent from learned information. In the following sections, we discuss some implications for neuropsychological sequelae of ALS. We also discuss the potential nosological specificity of computationally derived latent variables, and more generally some of the implications of the computational approach to neuropsychological assessment. Lastly, we outline study limitations and future research directions.

### 4.1. Implications for Neuropsychological Sequelae of ALS

Since ALS pathophysiology [15,17] comprises prefrontal cortical areas in a non-negligible subset of patients [22,23,24,25], we predicted that ALS patients may show evidence for alterations in latent variables of cognitive (i.e., putatively cortical) learning [41,79,101]. In line with this prediction, computational modeling of cWCST behavior revealed strong evidence for increased cognitive retention rates in ALS patients when compared to HC participants (see Figure 4 and Table 2). Please note that there was no evidence for any differences between ALS patients and HC participants with regard to neuropsychological characteristics as assessed by the Edinburgh Cognitive and Behavioural ALS Screen (ECAS) [117] (see Table A1).

**Figure 5:**
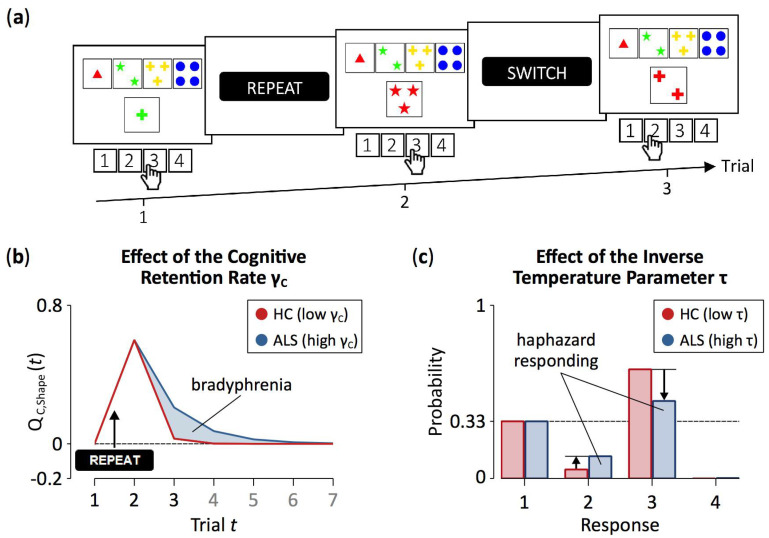
Exemplary effects of between-group variations of latent variables. Panel (**a**) shows the exemplary trial sequence on the cWCST as depicted in Figure 1. Panel (**b**) shows cognitive-learning feedback prediction values (referred to in the text as activation levels of objects of thought) for the application of the shape category across seven trials, the first three of them are shown in (**a**). The received positive feedback for the application of the shape category on Trial 1 causes an increase in feedback prediction values. With high configurations of the cognitive retention rate (i.e., $\gamma_{C}$), such as seen in ALS patients, feedback prediction values retain higher levels of activation from trial to trial. Note that the shape category was not applied on Trials 4 to 7. Panel (**c**) shows response probabilities on Trial 3. The probability of executing response 3 is highest (i.e., application of the shape category), followed by the probability of executing response 1 (i.e., application of the color category) and the probability of executing response 2 (i.e., application of the number category). With high configurations of the inverse temperature parameter (i.e., τ), such as seen in ALS patients, differences between response probabilities are attenuated, biasing response probabilities towards a uniform probability of 0.33. Note that the probability of executing an odd response (i.e., responses that match no viable sorting category; e.g., response 4 on Trial 3) is close to zero on any trial. Because the response probabilities of odd responses are virtually zero, such probabilities remain mostly unaffected by the inverse temperature parameter. The presented effects of model parameters were computed by varying exclusively the parameter of interest at arbitrary values while holding all other model parameters constant.

ALS patients showed increased cognitive retention rates when compared to HC participants, indicating that ALS patients retained more learned cognitive information from previous trials (see Figure 4). With higher cognitive retention rates, objects of thought (i.e., categories on the cWCST) retain higher activation levels from trial-to-trial (for illustration, see Figure 5a,b). These higher retention rates support adequate cWCST behavior in response to positive feedback, since the to-be-repeated categories remain at high levels of activation on upcoming trials. In contrast, high retention rates interfere with shifting the applied categories in response to negative feedback, since outdated categories exert stronger proactive interference on upcoming trials. Thus, flexibility of cognitive learning is reduced in ALS patients compared to HC participants. On the other hand, HC participants’ configurations of the cognitive retention rate might support adequate cWCST behavior in response to both, positive and negative feedback. We conclude that ALS pathophysiology [21] is associated with a latent cognitive symptom which can probably be best referred to as bradyphrenia (i.e., ‘inflexibility of thought’) [41,89]. The evidence for bradyphrenia in ALS patients corroborates the assumption that ALS should not merely be considered as a motor neuron disease [22,25,28]. Instead, a number of cognitive dysfunctions appear as a corollary of ALS pathophysiology [118,119,120].

Bradyphrenia is typically considered as a hallmark cognitive symptom of PD [89]. As such, our previous computational study [41] revealed evidence for bradyphrenia in PD patients, i.e., increased cognitive retention rates. However, results of the present study suggest that bradyphrenia is not specifically related to PD. In contrast to this common opinion, bradyphrenia should be conceived as a disease-nonspecific symptom, which occurs as a functional corollary of pathophysiological alterations in both PD and ALS patients. Computational cognitive neuropsychology provides an explicit definition of bradyphrenia at the level of covert cognitive processes that support cognitive flexibility (i.e., high retention of the activation of objects of thought) along with a novel computational indicator of bradyphrenia (i.e., increased cognitive retention rates). Thus, our computational modeling analysis revealed that both ALS and PD patients can be characterized at the level of covert cognitive processes by an increased retention of the activation of objects of thought (i.e., bradyphrenia) [41].

Computational modeling also revealed that ALS patients show increased inverse temperature parameters in comparison to HC participants. The inverse temperature parameter expresses how well finally executed responses corresponded to the information that has been learned from feedback which was received on previous trials (for illustration, see Figure 5a,c) [71,85,86,87]. With high values of the inverse temperature parameter, overt responses are more independent of learned information, rendering responding more haphazard. Thus, our results indicate that ALS pathophysiology [21] comprises another symptom, which might be best described as haphazard responding. Haphazard responding in ALS patients could also be related to subclinical manifestations of frontal disinhibition, which represents a major symptom of frontotemporal dementia [100,103]. Alternatively, it also remains possible that the increased inverse temperature parameter in ALS patients expresses ALS-related motor impairments, such as deficient fine motor skills that compromise responding as required for successful cWCST behavior [100].

### 4.2. Computational Modeling Provides Nosologically Specific Indicators of Executive Dysfunctions

**Figure 6:**
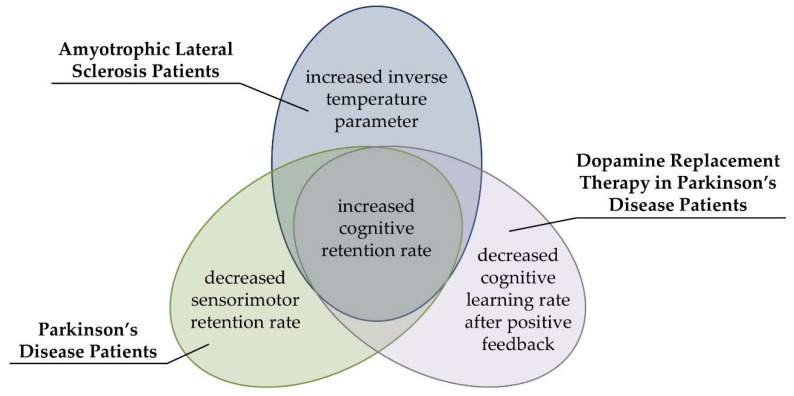
Patterns of alterations in latent variables of the parallel RL model as revealed by the present study and our previous computational study in PD [41] (see the text for details).

There was no evidence for alterations in conditional PE or SLE probabilities in ALS patients (see Table 1), and there was no evidence for such alterations in PD patients in our previous study [41]. Thus, ALS patients were phenomenologically indiscernible from PD patients with regard to analyses of behavioral cWCST error measures. Computational modeling [40] revealed that ALS and PD patients’ [41] likewise showed evidence for increased cognitive retention rates when compared with their respective HC participants. Thus, both ALS and PD pathophysiology seems to comprise a disease-nonspecific latent cognitive symptom, i.e., bradyphrenia (for illustration, see Figure 6) [41,89]. In addition to this common symptom, there were also differences between ALS and PD patients [41] in other latent variables of the parallel RL model [40]. ALS patients showed increased inverse temperature parameters when compared to HC participants (see Figure 4), whereas PD patients showed decreased sensorimotor retention rates when compared to HC participants in a related publication [41]. Furthermore, DA replacement therapy in PD patients decreased cognitive learning rates after positive feedback in that previous publication [41]. Thus, our data suggest that computational modeling by means of the parallel RL model [40] provides shared disease-related, and also nosologically specific alterations in latent variables in ALS and PD patients.

Our results corroborate that behavioral WCST measures, such as PE and/or SLE rates, do not provide sufficient nosological specificity to serve as pathognomonic neuropsychological symptoms of particular neurological diseases or psychiatric disorders [12,39]. We proposed that the insufficient nosological specificity of these traditional WCST error measures may be related to their ‘impureness’ [11,12,40,41,42,43]. That is, behavioral WCST measures may originate from a mixture of multiple covert cognitive processes, and diverse patient groups might show differentiable impairments in covert cognitive processes. However, such cognitive impairments may not be detectable via WCST error measures because they may all be behaviorally expressed as enhanced PE and/or SLE rates. In line with this assumption, computational modeling of covert cognitive processes by means of the parallel RL model [40] revealed that ALS and PD patients show disease-nonspecific increased cognitive retention rates (indicating bradyphrenia), but also disease-specific alterations in other latent variables (see Figure 6). We conclude that ALS and PD patients can be characterized by a mixture of shared and specific impairments at the level of covert cognitive processes, and we suggest that computational modeling provides an appropriate technique to complement studies of disease-related cognitive impairments.

The question of how latent variables of the parallel RL model [40] could be specifically related to some of the pathophysiologic characteristics of ALS and PD deserves further inquiry. Cognitive learning, as implemented in the parallel RL model [40], is assumed to be supported by prefrontal cortical areas of the brain [41,79]. As such, dysfunctions in prefrontal cortical areas might manifest as altered latent variables of cognitive learning. We found here and in our previous publication [34] that cognitive retention rates were increased in ALS and PD patients (see Figure 6). Thus, bradyphrenia represents a disease-nonspecific latent symptom, which may be related to to-be-delineated shared pathophysiological aspects of both diseases (ALS and PD).

ALS pathophysiology comprises prefrontal cortical areas in a non-negligible subset of patients [22,23,24,25]. More precisely, there is increasing evidence that ALS pathophysiology expands to the premotor cortex (PMC; Brodmann areas 6, 8, and 9) as well as to the dorsolateral prefrontal cortex (DLPFC; Broadman areas 46 & 9) [22,25]. In PD patients, the loss of DA neurons is most severe in nigro-striatal pathways [29,30] but other DA systems, such as the meso-cortical system, appear to be affected as well [121]. Importantly, the meso-cortical system is assumed to be overstimulated by DA replacement therapy in at least some PD patients [96,97,98,99]. The meso-cortical DA system comprises—among other cortical areas—the PMC and the DLPFC [122], so that these two cortical areas seem to be affected in PD patients as well as by PD patients’ DA replacement therapy. Thus, we hypothesize a correlation between increased cognitive retention rates and potential dysfunctions in the PMC and/or the DLPFC in both diseases that we consider here. This hypothesized correlation also stresses the notion that cognitive learning, as implemented in the parallel RL model [40], may be supported by prefrontal cortical areas of the brain [41,79,101]. Moreover, our data suggest that bradyphrenia should not be considered in a disease-specific manner, but that bradyphrenia might provide a marker of prefrontal dysfunctions in general.

There were some striking divergences between alterations in latent variables in ALS and PD patients (see Figure 6). First, inverse temperature parameters were found to be increased in ALS patients, but they were not found to be altered in PD patients [41]. Thus, increased inverse temperature parameters could be related to dysfunctions in the motor cortex of ALS patients [21]. Second, PD patients showed decreased sensorimotor retention rates, which indicated impaired stimulus-response learning in PD patients [41]. The sensorimotor retention rate was not found to be altered in ALS patients. Sensorimotor learning is putatively supported by striatal areas [41,79], which are primarily affected in PD patients [29,30]. Hence, decreased sensorimotor retention rates could be specifically related to PD patients’ dysfunctions in striatal brain areas. Finally, DA replacement therapy in PD patients induced decreased cognitive learning rates following positive feedback [41]. DA replacement therapy overstimulates the meso-limbic DA system in PD patients [96,97,98,99], which is associated with anticipation of reward or positive feedback [123,124]. Decreased cognitive learning rates following positive feedback due to DA replacement therapy might be a computational corollary of potential overstimulation of the meso-limbic DA system in PD patients through clinically titrated DA replacement therapy [41].

As an interim conclusion, latent variables of the parallel RL model [40] could be specifically related to some of the pathophysiologic characteristics of ALS and PD. The comparative analysis of ALS and PD patients’ alterations in latent computational variables remains correlational and should be interpreted with caution. However, this comparative analysis led to testable neuroanatomical hypotheses. In particular, the proposed relationship between cognitive retention rates and the prefrontal cortical areas should be addressed by confirmatory brain imaging studies. These WCST-based imaging studies should combine computational modeling with brain imaging [108], such as diffusion-weighted magnetic resonance imaging for the detection of disease-related micro-structural changes in brain areas of interest [125], or lesion-(latent) symptom mapping [62]. Such brain imaging studies should also address potential relationships of latent variables and other brain areas, which appear to be affected in ALS patients. For example, several studies suggest that dysfunctions in frontotemporal brain areas [24,126,127] and the basal ganglia [128] are related to executive dysfunctions in ALS patients.

### 4.3. Implications for Neuropsychological Assessment

The neuropsychological assessment of cognitive dysfunctions is almost entirely based on behavioral observations [12], with counting the occurrence of particular behavioral events, such as the number of PE and/or SE committed in the context of the WCST, as the current state-of-the-art approach to neuropsychological assessment. Such counts serve as indicators for the degree to which particular cognitive dysfunctions are present. For example, typical inferences from the presence of enhanced PE rates on the WCST would be that the assessed participant shows signs of cognitive inflexibility [4,9,10,11,40]. There are three shortcomings of this procedure [41]. First, neuropsychological assessment refers to cognitive assessment, yet the referenced cognitive processes remain unobservable. Thus, neuropsychological assessment involves inferences that go beyond behavioral observations. Second, cognitive constructs, which are referred to by neuropsychological assessment, are often vaguely defined. That is, cognitive constructs are typically verbal re-descriptions of the behavioral observations that were made (take PE as an indicator of cognitive inflexibility as an example). Moreover, the referenced behavioral observations may not originate from a single cognitive process but should better be conceived as resulting from a mixture of multiple covert cognitive processes [11,12,40,41,42,43]. Finally, observable behavioral measures on neuropsychological assessment instruments do not possess satisfactory nosological specificity for diverse patient groups, as detailed above [12,39].

Computational cognitive neuropsychology offers a route towards advanced neuropsychological assessment, which could remedy some of these shortcomings. Computational modeling offers a technique to decompose behavior on neuropsychological assessment instruments into assumed latent cognitive processes, allowing inferences closer to the level of covert cognitive processes [41,46]. Importantly, latent variables, which reflect the efficacy of covert cognitive processes, are unambiguously defined (see Appendix B) and, thereby, may replace the traditional verbal constructs of neuropsychological assessment [47,48,49,50,51,52,53,54,55,56,57]. The present study exemplifies that latent variables obtained from computational modeling may provide indicators of shared latent symptoms as well as nosologically specific differentiable facets of latent executive dysfunctions. Thus, computational cognitive neuropsychology offers the potential to improve inferential validity of neuropsychological assessment.

### 4.4. Study Limitations and Directions for Future Research

In line with previous studies [40,41], estimates of the sensorimotor learning rate after positive feedback were virtually zero (see Figure 4), indicating that sensorimotor learning did not happen following positive feedback (for a detailed discussion, see [40]). However, there was strong evidence for an effect of group membership on the sensorimotor learning rate after positive feedback (see Table 2). Inspection of Figure 4 revealed that this evidence for a group effect on the sensorimotor learning rate following positive feedback was most likely due to one single outlier in the ALS patients. Thus, we did not interpret the evidence for a group effect on this close-to-zero model parameter.

A recent meta-analytical review [15] reported evidence for enhanced PE rates in ALS patients. In the present study, mean conditional PE probabilities were increased in ALS patients when compared to HC participants (see Figure 3). However, this increase in conditional PE probabilities was not supported by any evidence from the Bayesian ANOVA (see Table 1). This missing evidence was most likely due to the small sample size typical for neuropsychological ALS studies [129], which constitutes a limitation of this study.

There are two differences between the cWCST variant that we utilized in the present study and the cWCST variant that was used to characterize PD patients’ alterations in latent variables of the parallel RL model [41]. First, in the present study, the cWCST was completed after a fixed number of 120 trials. In contrast, the cWCST variant that was utilized to assess PD patients ended after a fixed number of 40 completed categories irrespective of the number of completed trials (median = 212 trials, min = 170 trials, max = 281 trials). Second, in this study, the prevailing sorting category switched after a random number of trials, which was defined prior to the experiment. On the cWCST variant that was utilized to assess PD patients, participants had to complete a minimum of two correct card sorts to trigger a switch of the prevailing sorting category. However, estimates of the latent variables of interest (i.e., latent variables that were alternating between patient groups and healthy controls) were comparable in size between the current study of ALS patients and the previous study of PD patients [41]. We conclude that any differences between the administered cWCST variants do not interfere with comparisons of alterations in latent variables of ALS and PD patients. However, future research should address how specific configurations of the cWCST could affect parameter estimates of the parallel RL model.

We assessed neuropsychological characteristics of ALS patients and HC participants by means of the ECAS [117]. The ECAS allows for the assessment of both ALS-specific and ALS-nonspecific neuropsychological characteristics with strong clinical validity [130]. However, we did not find evidence for any differences between ALS patients and HC participants with regard to the ECAS (see Table A1). The missing evidence was most likely due to the small sample size, which is typical for neuropsychological ALS studies [129]. Alternatively, a more extensive evaluation of the neuropsychological characteristics of ALS patients and HC participants may have been required.

In a recent study [40], we demonstrated that the parallel RL model provides a better conceptualization of behavioral cWCST data than competing computational models [42]. This conclusion was based on the analysis of a large sample of young volunteers (*N* = 375). It remains to be seen whether this finding transfers to other populations, such as ALS patients. As the sample size was relatively small in the present study, we did not consider any model comparisons. However, future studies should test which of all applicable computational models provides the best conceptualization of the to-be-studied behavioral data and base further analyses on the winning computational model [131].

## 5. Conclusions

Computational modeling of cWCST behavior revealed specific facets of executive dysfunction in ALS patients, which are difficult to study by traditional WCST error measures. Our data indicate ALS-related latent cognitive symptoms, which might be best referred to as bradyphrenia and as haphazard responding. Bradyphrenia seems to represent a disease-nonspecific latent cognitive symptom in ALS and PD patients, but disease-specific latent cognitive symptoms were also discernible. Thus, our results suggest that latent variables of the parallel RL model [40] provide nosologically specific indicators of latent facets of executive dysfunction in ALS and PD patients. The comparative analysis of ALS and PD patients’ alterations in latent dysexecutive symptoms resulted in novel hypotheses about which brain areas support these specific facets of executive dysfunction, paving the way for future confirmatory brain imaging studies.

## Figures and Tables

**Table 1:** Analysis of effects of error type and group on conditional error probabilities.

Effects	*P*(Inclusion)	*P*(Inclusion|Data)	BF_inclulsion_
Error Type	0.600	0.999	836.42 **
Group	0.600	0.656	1.27
Error Type x Group	0.200	0.385	2.50

** extreme evidence.

**Table 2:** Bayes factors for effects of group on model parameters.

Parameter	Description	Bayes Factor
$\alpha_{C}^{+}$	cognitive learning rate following positive feedback	0.81
$\alpha_{C}^{-}$	cognitive learning rate following negative feedback	0.34
$\gamma_{C}$	cognitive retention rate	37.46 *
$\alpha_{S}^{+}$	sensorimotor learning rate following positive feedback	14.96 *
$\alpha_{S}^{-}$	sensorimotor learning rate following negative feedback	0.44
$\gamma_{S}$	sensorimotor retention rate	1.49
τ	inverse temperature parameter	17.07 *

* strong evidence.

## Appendix A

**Table A1:** Demographic, clinical, and psychological characteristics.

		HC (*N* = 21)			ALS (*N* = 18)			Bayes Factor
	Maximum	Mean	*SD*	*n*	Mean	*SD*	*n*	
Age (years)		57.67	9.16	21	58.11	10.05	18	0.32
Female [%]		22%			29%			0.91 ^#^
Education (years)		14.29	2.93	21	13.92	2.16	18	0.34
Disease Duration (months)					50.47	82.93	17	
FVC					85.75	12.84	16	
ESS	24				6.12	3.06	17	
ALSFRS-EX: total	60				47.41	6.99	17	
Bulbar Subscore	16				13.94	1.89	17	
Fine Motor Subscore	16				11.41	2.87	17	
Gross Motor Subscore	16				11.12	5.02	17	
Respiratory Subscore	16				10.94	1.71	17	
Progression Rate	16				0.71	0.57	17	
MoCA	30	27.24	2.68	21	26.72	3.18	18	0.35
FAB	18	17.19	1.25	21	16.50	2.19	16	0.57
ECAS: total	136	104.38	11.58	21	103.89	12.19	18	0.32
ECAS: ALS-Specific	100	75.48	10.63	21	75.89	10.26	18	0.40
Language	28	26.62	1.94	21	25.94	2.13	18	0.48
Fluency	24	11.52	5.83	21	10.89	4.13	18	0.33
Executive	48	37.33	4.50	21	39.06	5.92	18	0.48
ECAS: ALS-Nonspecific	36	28.86	3.07	21	28.00	3.66	18	0.31
Memory	24	17.24	2.83	21	16.44	3.54	18	0.40
Visuospatial	12	11.62	0.74	21	11.56	0.62	18	0.32

Bayes factors were obtained from Bayesian independent samples *t*-tests. ^#^ Bayes factor was obtained from Bayesian contingency tables. All inferential statistics were carried out by means of JASP [106]. ALS = Amyotrophic lateral sclerosis patients; HC = healthy control participants; FVC = forced vital capacity; ESS = Epworth Sleepiness Scale [132]; ALSFRS-EX = Amyotrophic Lateral Sclerosis Functional Rating Scale—Self-Rating Scale [133]; MoCA = Montreal Cognitive Assessment [134]; FAB = Frontal Assessment Battery [135]; ECAS = Edinburgh Cognitive and Behavioural ALS Screen [117]; Please note that the present study utilized different WCST-based inclusion criteria for ALS patients and HC participants when compared to the initial publication of the data [15].

## Appendix B

The cognitive Q-learning algorithm operates on feedback prediction values for the application of sorting categories. $Q_{C}\left(t\right)$ is a 3 (categories) × 1 vector that quantifies feedback prediction values for the application of sorting categories on trial *t*. The strength of transfer of feedback prediction values from trial to trial is modeled as:

(A1) $$Q_{C}^{\prime }\left(t\right)=\gamma_{C}*Q_{C}\left(t\right)$$

with $\gamma_{C}$ is the individual cognitive retention rate. Trial-wise cognitive prediction errors $\delta_{C}\left(t\right)$ are computed with regard to the applied category *u* ∈ *U* on trial *t* as:

(A2) $$\delta_{C}\left(t\right)=r\left(t\right)-Q_{C,u}^{\prime }\left(t\right)$$

*r*(*t*) denotes the received feedback on trial *t*, which is 1 for positive feedback and −1 for negative feedback. Feedback prediction values for the application of sorting categories are updated as:

(A3) $$Q_{C}\left(t+1\right)=Q_{C}^{\prime }\left(t\right)+Y_{C}\left(t\right)*\alpha_{C}*\delta_{C}\left(t\right)$$

$Y_{C}\left(t\right)$ is a 3 (categories) × 1 dummy vector, which is 1 for the applied category *u* and 0 for all other categories on trial *t*. The dummy vector $Y_{C}\left(t\right)$ ensures that only the feedback prediction value for the applied category *u* on trial *t* is updated by the cognitive prediction error $\delta_{C}\left(t\right)$. $\alpha_{C}$ denotes the cognitive learning rate which was further separated for trials following positive and negative feedback (i.e., $\alpha_{C}^{+}$ and $\alpha_{C}^{-}$, respectively).

Feedback prediction values for the application of categories $Q_{C}\left(t+1\right)$ are matched to responses, which is represented by a 4 (responses) x 1 vector $Q_{RC}\left(t+1\right)$. For response *v* ∈ *V* on trial *t* + 1, $Q_{RC,v}\left(t+1\right)$ is computed as:

(A4) $$Q_{RC,v}\left(t+1\right)=X_{v}^{T}\left(t+1\right)\;Q_{C}\left(t+1\right)$$

with $X_{v}\left(t+1\right)$ is a 3 (categories) × 1 vector that represents the match between a stimulus card and response *v* with regard to sorting categories. Let $X_{v}\left(t+1\right)$ be 1 if a stimulus card matches response *v* by category *u* and let $X_{v}\left(t+1\right)$ be 0 elsewise. $X_{v}^{T}\left(t+1\right)$ is the transpose of $X_{v}\left(t+1\right)$. To account for responses that match no viable sorting category (i.e., responses that certainly yield a negative feedback with regard to cognitive learning), feedback prediction values of such responses are assigned a value of $Q_{RC,v}\left(t+1\right)=-1$.

The sensorimotor Q-learning algorithm parallels the cognitive Q-learning algorithm. However, the sensorimotor Q-learning algorithm directly operates on feedback prediction values for the execution of responses. A 4 (responses) × 1 vector $Q_{S}\left(t\right)$ quantifies feedback prediction values for the execution of responses on trial *t*. The strength of the transfer of feedback prediction values from trial to trial is given by:

(A5) $$Q_{S}^{\prime }\left(t\right)=\gamma_{S}*Q_{S}\left(t\right)$$

$\gamma_{S}$ denotes the individual sensorimotor retention rate. The sensorimotor prediction error $\delta_{S}\left(t\right)$ on trial *t* is computed as:

(A6) $$\delta_{S}\left(t\right)=r\left(t\right)-Q_{S,v}^{\prime }\left(t\right)$$

Here, $Q_{S,v}^{\prime }\left(t\right)$ is the feedback prediction value of executed response *v* on trial *t*. Feedback prediction values for the execution of responses are updated as:

(A7) $$Q_{S}\left(t+1\right)=Q_{S}^{\prime }\left(t\right)+Y_{S}\left(t\right)*\alpha_{S}*\delta_{S}\left(t\right)$$

Again, $Y_{S}\left(t\right)$ denotes a 4 (responses) × 1 dummy vector that is 1 for the executed response *v* and 0 for all other responses on trial *t*. $Y_{S}\left(t\right)$ ensures that only feedback prediction values of the executed response are updated by the sensorimotor prediction error $\delta_{S}\left(t\right)$. We assumed independent individual sensorimotor learning rate parameters for positive and negative feedback, $\alpha_{S}^{+}$ and $\alpha_{S}^{-}$, respectively.

Feedback prediction values of cognitive and sensorimotor Q-learning algorithms are linear integrated. More precisely, integrated feedback prediction values $Q_{sum}\left(t+1\right)$ for trial *t* + 1 are computed as:

(A8) $$Q_{sum}\left(t+1\right)=Q_{RC}\left(t+1\right)+Q_{S}\left(t+1\right)$$

The response probability $P_{v}\left(t+1\right)$ of response *v* on trial *t* + 1 is derived from integrated feedback prediction values using a “softmax” logistic function [71]:

(A9) $$P_{v}\left(t+1\right)=\frac{e^{\frac{Q_{sum,v}\left(t+1\right)}{\tau }}}{\sum_{j=1}^{4}e^{\frac{Q_{sum,j}\left(t+1\right)}{\tau }}}$$

with τ denotes the individual inverse temperature parameter.

Parameters of the parallel RL model (i.e., $Z\in \left[\alpha_{C}^{+},\alpha_{C}^{-},\gamma_{C},\alpha_{S}^{+},\alpha_{S}^{-},\gamma_{S},\tau \right]$) were estimated using hierarchical Bayesian analysis [111,136,137,138,139,140,141,142] by means of RStan [143]. Hierarchical Bayesian analysis allows for individual differences in model parameters while pooling information across all individuals by means of hyper- (i.e., group-level) parameters [84,136,138]. More precisely, any individual-level model parameter $z_{i}$ of participant *i* was drawn from a hyper-level normal distribution with mean $\mu_{z}$ and standard deviation $\sigma_{z}$. In order to facilitate hierarchical Bayesian analysis, we implemented a non-centered parameterization [111,144]. That is, individual-level model parameters were sampled from a standard normal distribution, which was multiplied by a hyper-level scale parameter $\sigma_{z}$ and shifted by a hyper-level location parameter $\mu_{z}$. The individual deviation of model parameter $z_{i}$ of participant *i* from $\mu_{z}$ was modeled by an individual-level location parameter $z\prime_{i}$ [40,41]. Taken together, model parameter $z_{i}$ of participant *i* was implemented as:

(A10) $$z_{i}=\mu_{z}+\sigma_{z}*{z^{\prime }}_{i}$$

In order to account for effects of ALS pathology on model parameters, we introduced the between-subjects effect group [41,108,145]. That is, group-related shifts of model parameters were implemented by adding the hyper-level parameter $\mu_{group,z}$ to the hyper-level location parameter $\mu_{Z}$ for all HC participants. Please note that we implemented the hyper-level parameter $\mu_{group,z}$ for all HC participants rather than for all ALS patients because the sample size was larger for HC participants, which allowed for estimation of $\mu_{group,z}$ with higher statistical power.

We further facilitated hierarchical Bayesian analysis by conducting parameter estimation in an unconstrained space [40,41,111,137,144]. That is, all location and scale parameters were free to vary without any constraints. The linear integration of location and scale parameters was then mapped to a constrained space (i.e., [0, 1]) using the Probit function. The Probit function is the inverse-cumulative standard normal distribution. Taken together, individual-level model parameter $z_{i}$ for any ALS patient was modeled as:

(A11) $$z_{\;i}=Probit\left(\mu_{z}+\sigma_{z}*{z^{\prime }}_{i}\right)$$

and individual-level model parameter $z_{i}$ of any HC participant was modeled as:

(A12) $$z_{HC,i}=Probit\left(\mu_{z}+\sigma_{z}*{z^{\prime }}_{i}+\mu_{group,z}\right)$$

In line with previous research, we assumed normal prior distributions (*μ* = 0, *σ* = 1) for location parameters and half Cauchy prior distributions (*μ* = 0, *σ* = 5) for scale parameters [40,41,111]. Parameter estimation was done using three chains of 1000 iterations, including 500 warm-up iterations each. Parameter estimation was inspected quantitatively by the $\hat{R}$ statistic [146] and the effective sample size statistic as well as visually by trace-plots. Feedback prediction values of the parallel RL model were initialized as 0.

We used Bayesian tests for direction to quantify evidence for group-related shifts of model parameters [108,115]. For any model parameter *z*, we computed a Bayes factor (BF) by dividing the posterior density of parameter $\mu_{group,z}$ below zero by the posterior density above zero. Please note that Bayesian tests were applicable, as prior distributions of $\mu_{group,z}$ were symmetric around zero [147].

Descriptive statistics of model parameters were obtained from posterior distributions of hyper-level location parameters. More precisely, the posterior distribution of model parameter *z* of ALS patients was given by:

(A13) $$z_{ALS}=Probit\left(\mu_{z}\right)$$

For HC participants, the posterior distribution of model parameter *z* was given by:

(A14) $$z_{HC}=Probit\left(\mu_{z}+\;\mu_{group,z}\right)$$

Descriptive statistics of posterior distributions were reported as medians with lower and upper quartiles. We computed individual-level parameter estimates as medians of individual-level posterior distributions as derived by Equations (A11) and (A12). Correlational analysis was done using JASP version 0.11.1 [106]. The implemented code is available from https://osf.io/46nj5/.
